# Medical Extracts—Practical and Philosophical

**Published:** 1873-09

**Authors:** L. B. Anderson

**Affiliations:** Virginia


### MEDICAL EXTRACTS — PRACTICAL AND PHILOSOPHICAL.

BY L. B. ANDERSON, M.D., OF VIRGINIA.

### *MEDICAL PHILOSOPHY.*

The first step in the study of disease, requires a thorough knowledge of the anatomical characteristics of the organs of organic life. The next is a clear appreciation of the nature and vital importance of the functions of those organs. In this last is involved, not only the peculiar nature of the function *per se*, and its direct influence upon vitality, but also its relative influence upon the functions of other organs. A perversion of the renal function might exert a very deleterious influence on the vital functions; but that may be only a sequence of a prior and more serious disorder of the hepatic function. Hence, it is as essential to know the relationship existing between the functions of different organs, as it is to understand the influence exerted by one organ on the economy as a whole. Equally essential is a knowledge of the generation and purification of the blood by a harmonious functional activity, the forces which circulate it, and finally, how both are promoted, modified or perverted, by varying conditions of the nervous system.

Having acquired these essential and fundamental facts, we will be prepared to follow nature into her workshop and watch her efforts, sometimes successful, often fruitless, in resisting the assaults of morbific agents upon the economy. We may see how the function of one organ being perverted, or the circulation in one portion of the capillaries being deranged, the whole mass of vital fluid is thrown from its balance and fever is produced. But most important of all, we will be able to comprehend and appreciate the true nature and philosophy of inflammation, the nidus from which nearly all the ills to which our flesh is heir, have sprung. The *modus operandi* of the changes which are wrought in the muscular system, and the concomitant nervous participation in the incubation, passivity and maturation of inflammation, and its terminations in atrophy, hypertrophy, induration, softening, morbid growths, effusions, suppuration, resolution and gangrene, constitute the key by which we may unlock the hidden operations of nature, and view them from the aberration of the first truant cytoblast in an obscure capillary, to full development of wasting disease, or a return to health.

Benignant nature has kindly supplied the human system with windows, as it were, through which the observant physician may look in and view, with almost undimmed vision, the morbid changes which are effected in the citadel of life, during the progress of disease. The changes in temperature, the character of the pulse, the appearance of the tongue, the sputa, alvine evacuations, the urine, the skin, the sounds emitted from the heart and lungs, the condition of the abdomen, the character and odor of the perspiration, renal, intestinal and pulmonary evacuations, and uterine discharges—the posture, appetite, breathing, vision, hearing, the sensations, and last, though not least, the countenance, are so many lenses through which the discriminating physician may look in and view the struggles of the vital organs, as they toil beneath the incubus of dire disease. So expressive is the countenance of the true state and condition of the system in many diseases, that we require but slight collateral evidence to read, with unerring certainty, the lines which are traced on its lineaments. A slight touch of the pulse and a glance at the posture of a parturient woman, enables us to read in the constrained, contracted and anxious features the unmistakable evidence of puerperal peritonitis. The open mouth, dilated nostrils and wistful eye, with slight pulmonary crepitus and hurried breathing, in a helpless infant, point with unerring certainty to the existence of pneumonia. In the dilated pupils, expanded and sluggish lids, shrunken features, peculiar whine and anxious, restless countenance, we read the lesson of exhausted brain-power in a teething child. Few, indeed, are the diseases to which our flesh is heir, which have not recorded some line peculiar to itself, upon the physiognomy of the sufferer.

So beautifully arranged are the organs of the human system, that their functions are made mutually dependent and coöperative. Impair the ærating functions of the lungs, and the liver assumes the responsibility of separating the carbonaceous constituents from the blood, which should have been eliminated by them. If the liver be unable to meet the demands thus made upon it, the kidneys come to the rescue and industriously ply their energies to relieve the oppressions of the laboring organism. In increasing the exigencies of one of these organs, the troubles and embarrassments of the others are proportionally diminished. This sympathy and coöperation often affords an opportunity of giving relief, even when the functions of a vital organ have been seriously and permanently impaired.

Interesting and profitable as is the study of man, in his physical organism, were our studies and investigations to be confined to, and circumscribed by, the functions of organic life, there would hang over our physiological and pathological outlook a mist and darkness which would render the practice of medicine a mere gamble for human life, which would make our labors as unsatisfactory to ourselves as unprofitable to our patients; for, in the very perfection of the operation of the laws of organic life, a new principle is brought into being, characterized by affinities and repulsions which demand our closest study and most constant attention—affinities and repulsions which are ever present to aid or foil the physician, as he permits them to operate to his injury, or induces them to conspire to his profit. This principle, though common to the whole animal kingdom, and even faintly foreshadowed in the higher orders of plants, attains its highest perfection only in the human species; but in whomsoever or in whatsoever its characteristics are developed, we call it Life. "Life," says the great Bechat, "is the totality of those functions which resist death:" that is, the combined operation of the functions of the animal economy develop a principle which we call life.; which principle is in its highest degree of development when those functions are performed with the greatest energy and harmony. And it deteriorates and degenerates in the direct ratio that those functions are impaired. And when they cease altogether, it becomes extinct. This animal development, or life, while a creature of mere organic energy, renders the animal organism the object and recipient of thousands of impressions to energize its functions, paralyze its powers, or waste its energies. The outer world being thus brought into direct contact with the inner organism, through the principle of life, all objects become pleasing or painful, healthful or hurtful, as they produce harmony or discord in the body. Thus, lust, fear, love, hatred, appetite or instinct are produced in accordance with the affinities or repulsions of the organism. Phenomena outside of, and entirely distinct from, the physical being, through the reflex influence of vital force, may thus conduce to health and vitality, or disorganization and death. He who has failed to study the laws of animal life, and the relation they sustain to, and the influence they extend over, the functions of organic life, has yet to find the key which will disclose many of the mysteries which enshroud the nature of disease, as well as elucidate the *modus operandi* of many remedial agents.

As in the organic world, where all the operations of nature are controlled by the laws of affinity and repulsion, producing compounds of definite proportions and uniform principles, so the evolutions in organic life are maintained by like unchangeable, definite and uniform principles, producing results of the most unvarying characteristics. Hence, while the combining proportions of marble constituents are always the same, a difference in the physical qualities of the ultimate atoms of which it is composed, produces results as dissimilar in color and form, as those which characterize the human organism. In both kingdoms, like agents and laws produce like effects, and those agents and laws are as invariable and unchangeable as light and darkness. The characteristics and qualities of all bodies, whether inorganic or organic, depend upon the inherent properties of their germ atom or germ cell; the germ atom of marble will produce marble, of granite, granite—presenting, always and everywhere, the same uniform chemical composition, but differing in their physical properties according to the inherent qualities of their germ atom; which quality depends in no wise upon any adventitious or accidental circumstance, but owes its origin to the impress which it received while bright and new, it dropped from the fingers of Creation. Thus, the inherent quality of the germ cell of organic matter has developed in the human race, species as dissimilar as the colors of marble. The germ cell of the white man will always, and everywhere, produce a white man; of the Indian, an Indian; and of the curly-headed, thick-lip, flat-nose, muciferous-stench, black-skin African, has ever, and will ever, produce his counterpart in all ages and countries, and under all circumstances and conditions. Thus, the law of evolution presides over the germ cell of all organic matter, and the blood of man is identical to-day with that which coursed in the veins of Adam, producing organs and tissues absolutely and identically the same, and bearing the same relations and characteristics which they did in our first parent. The tiger of Bengal and the lion of Africa, the spots of the leopard and the skin of the Ethiopian are unchanged and unchangeable. After the lapse of three thousand years, with its accumulated lights and "progressive evolutions," Ulysses Grant is not to be compared with Alexander the Great, Sumner with Socrates, or Butler with Cicero. The liver, lungs and kidneys, the brain, uterus and testicles perform the same functions to-day they did in the days of Abraham. Hellebore and hemlock produce the same effects on the human organism they did in the days of Hypocrates. Hence, all the facts in anatomy, physiology, pathology, hygiene or therapeutics which were applicable to man in his physical or animal nature in the incipiency of our race, are equally applicable to him to-day.

Springing from the hand of God in all the perfection of a complete organism, the phenomena of animal life were developed in man, above all other animals, commensurate with the superiority, symmetry and beauty of his physical being; yet no instincts, emotions or aspirations had he above those of other animals. Wonderful as were the functions of his vital organs, and amazing as were the phenomena springing from their combined and harmonious action, man was only an animal—yet occupying the head of the column—with all the passions, emotions, appetites and instincts of the *mere* animal! The finishing touch was yet to be given, and man was to be endowed with a new principle—a spiritual nature, which should distinguish him from all mundane beings—a principle as immortal as God himself, springing from, and aspiring to, heaven, it was destined to bring his animal nature, with all its groveling passions and appetites, in subjection to it. To this end, "God breathed into his nostrils the breath of life, and he became a living soul." The objects of time and sense were not henceforth to be only agents which were to operate on, and influence him; nor was he destined long to move in that spiritual atmosphere which now enveloped him; for, yielding in an evil hour to the cravings of his mere animal nature, his immortal principle, with all its hopes and aspirations, were brought into subjection to it, and man was destined to become the medium of impressions the most momentous and profound. The hopes and fears arising from his two-fold nature—the conflict between his animal and spiritual natures—the upbraidings of a guilty conscience, amid the indulgence of carnal appetites and sensual lusts—the depressions and disappointments which crowd upon the mind as busy memory brings up, in grim array, time mis-spent, opportunities neglected and words and acts performed and uttered, which can never be erased or recalled. All these become fruitful *materies morbi*, stealthily, but surely operating through the animal nature upon the organic functions, and claiming the nicest discrimination of the pathologist, as prying into the nature of disease, he seeks the indications of cure, combining in his physical organization almost every agent, and influenced by almost every law of the material universe; and by his two-fold nature united with two worlds, man becomes the centre of influences and agencies which spring alike from earth and heaven, the past and future, time and eternity. Wonderfully and fearfully made, indeed, is he; and that man who analyzes and comprehends the laws by which he is governed, and discovers and describes the agencies and influences which operate upon and control him, is a philosopher indeed.

In view of the complexity of the human constitution, the numerous agencies and influences which operate upon it, and the multiform ills to which it is heir, the mind is at first appalled at the idea of assuming the responsibility of diagnosing its diseases and rectifying its disorders. In sweeping, however, over the long drawn catalogue of human diseases, we will find that busy hands have been at work during all the ages, collecting and arranging facts and deducing general principles, which gleam as beacon-lights to direct the devotee of science through its intricate pathways. Thus, diseases and remedies have been arranged in corresponding classes, which, enabling the physician to investigate and measure didactically and logically, reduces his labors to a pleasant toil, and his conclusions, in many instances, to a mathematical certainty. Of these facts and principles, these deductions and conclusions, we pro-propose to treat in a future number.

---

Ointment for Freckles.—℞. Flour of mustard, ℥iij.; lemon juice to make a stiff paste, q. s.; oil of almonds, ℥ss. Mix.

